# The use of clinical decision rules for pulmonary embolism in the emergency department: a retrospective study

**DOI:** 10.1186/s12245-020-00281-1

**Published:** 2020-05-11

**Authors:** Omran Al Dandan, Ali Hassan, Afnan Alnasr, Mohammed Al Gadeeb, Hossain AbuAlola, Sarah Alshahwan, Malak Al Shammari, Alaa Alzaki

**Affiliations:** 1 Department of Radiology, King Fahd Hospital of the University, Imam Abdulrahman Bin Faisal University, Al-Khobar, Saudi Arabia; 2 Department of Family and Community Medicine, Imam Abdulrahman Bin Faisal University, Dammam, Saudi Arabia; 3 Department of Internal Medicine, King Fahd Hospital of the University, Imam Abdulrahman Bin Faisal University, Al-Khobar, Saudi Arabia

**Keywords:** Computed Tomography, Pulmonary embolism, Wells criteria, Guidelines

## Abstract

**Background:**

Pulmonary embolism (PE) is a common and life-threatening medical condition with non-specific clinical presentation. Computed tomography pulmonary angiography (CT-PA) has been the diagnostic modality of choice, but its use is not without risks. Clinical decision rules have been established for the use of diagnostic modalities for patients with suspected PE. This study aims to assess the adherence of physicians to the diagnostic algorithms and rules.

**Methods:**

A retrospective observational study examining the utilization of CT-PA in the Emergency Department of King Fahd Hospital of Imam Abdulrahman Bin Faisal University for patients with suspected PE from May 2016 to December 2019. The electronic health records were used to collect the data, including background demographic data, clinical presentation, triage vital signs, D-dimer level (if ordered), risk factors for PE, and the CT-PA findings. The Wells score and pulmonary embolism rule-out (PERC) criteria were calculated retrospectively without knowledge of the results of D-dimer and the CT-PA.

**Results:**

The study involved a total of 353 patients (125 men and 228 women) with a mean age of 46.7 ± 18.4 years. Overall, 200 patients (56.7%) were classified into the “PE unlikely” group and 153 patients (43.3%) in the “PE likely” group as per Wells criteria. Out of all the CT-PA, 119 CT-PA (33.7%) were requested without D-dimer assay (*n* = 114) or with normal D-dimer level (*n* = 5) despite being in the “PE unlikely” group. Only 49 patients had negative PERC criteria, of which three patients had PE.

**Conclusions:**

The study revealed that approximately one-third of all CT-PA requests were not adhering to the clinical decision rules with a significant underutilization of D-dimer assay in such patients. To reduce overutilization of imaging, planned interventions to promote the adherence to the current guidelines seem imperative.

## Background

Pulmonary embolism (PE) is a common and potentially life-threatening medical condition. Every year, there are approximately 100,000 deaths from PE in the USA [1]. PE has a wide range of presenting symptoms ranging from no symptoms to sudden death. Due to its non-specific signs and symptoms, the diagnosis of PE is often missed or delayed [2]. Early diagnosis and management are crucial as the mortality rate reaches nearly 30% for untreated PE [3]. Computed tomography pulmonary angiography (CT-PA) has become the modality of choice in the evaluation of patients with suspected PE because of its high sensitivity and accuracy [4]. However, an increase in the use of computed tomography scans has become a subject of concern as it may subject patients to ionizing radiations and contrast-related complications and increase the hospital stay and expenditure [5–7]. Of note, it has been reported that the USA spends approximately twice as much as other high-income countries on the healthcare and overutilization of diagnostic imaging was the main reason [8].

Diagnostic algorithms and clinical decision rules have been established to guide in the management of patients with suspected PE. Such criteria include the Wells criteria and the pulmonary embolism rule-out criteria (PERC) which were found to have high predictive accuracy [9, 10]. In the modified Wells criteria, the patient who has a score ≤ 4 is considered unlikely to have PE and should have D-dimer testing. If the D-dimer level is normal, no further workup is warranted. The CT-PA is recommended in case of elevated D-dimer level or for Wells score > 4 [11]. Similarly, the patient who does not meet any of the PERC criteria should not have any further workup for PE [10]. The implementation of the diagnostic algorithms is essential and advocated by national guidelines [12, 13].

In Saudi Arabia, however, there has been no previous study investigating the adherence of physicians to the clinical decision rules for patients with suspected PE. Therefore, this study was designed to assess the adherence to these rules in the emergency department (ED).

## Methods

### Study design

We conducted a retrospective study examining the adherence of physicians to the clinical decision rules for PE among patients with suspected PE in the ED.

### Study setting

The study was conducted in King Fahd Hospital of the Imam Abdulrahman Bin Faisal University, which is a 440-bed capacity academic center and is located in the Eastern Province of Saudi Arabia. Approximately 250,000 patients visit the ED and 20,000 patients are admitted every year. In our institution, CT-PA can only be requested by board-certified consultant physicians.

### Study population

Eligible patients included all adults (≥ 18 years) with suspected PE who underwent CT-PA. The radiology information system was utilized to identify all CT-PA requests from the ED from May 2016 to December 2019.

### Data collection

A web-based structured data collection form was used to collect the data from the electronic health record. The collected data included background demographic data, clinical presentation, D-dimer level (if ordered), risk factors for PE (immobilization or surgery within past 4 weeks, previous deep venous thrombosis or PE, and exogenous estrogen use), and the CT-PA findings.

The imaging results were collected from the electronic reports for assessing the presence of pulmonary artery filling defects. In case the patient had more than one CT-PA scan, only the most recent was included in our study. The Wells and PERC scores were calculated retrospectively by investigators without the knowledge of D-dimer and CT-PA results. The patients were categorized into the “PE likely” group if the Wells score was > 4 and the “PE unlikely” group if the Wells score was ≤ 4.

### Imaging technique

The scans were performed from the diaphragm to the lung apices, using one of the two multidetector scanners:

1. The dual-source 128-slice scanner (Somatom Definition Flash, Siemens; Erlangen, Germany) with the administration of 25-ml non-ionic contrast material with timed intravenous pump infusion followed by a 25-ml saline flush, after the test bolus.
2. The 64-slice scanner (Somatom Definition AS, Siemens; Erlangen, Germany) with the administration of 60-ml non-ionic contrast material with timed intravenous pump infusion followed by a 40-ml saline flush, with bolus tracking.

### D-dimer assay

A rapid quantitative immunoturbidimetric assay (Advanced D-dimer; Dade Behring, Marburg, Germany) was used for the D-dimer assay. The results were expressed as mg/ml and the normal range was defined as < 500 mg/ml. The result was usually reported within 1 h.

### Statistical analysis

The obtained data were compiled using the QuestionPro platform and analyzed using IBM SPSS for Windows, version 25 (IBM Corp., Armonk, NY, USA). Continuous variables were presented as mean ± standard deviation. Descriptive statistics, such as percentages and frequency distribution of different characteristics, were used as appropriate. Categorical variables were compared using chi-square test. The statements of statistical significance were based on a significance level of *α* = 0.05.

## Results

### Patients characteristics

The study involved a total of 353 patients, including 125 men and 228 women, representing 35.4% and 64.6% of the study population, respectively. The mean age of patients was 46.7 ± 18.4 years (range, 18–96 years).

### Decision rules

#### Wells criteria

**Fig. 1:**
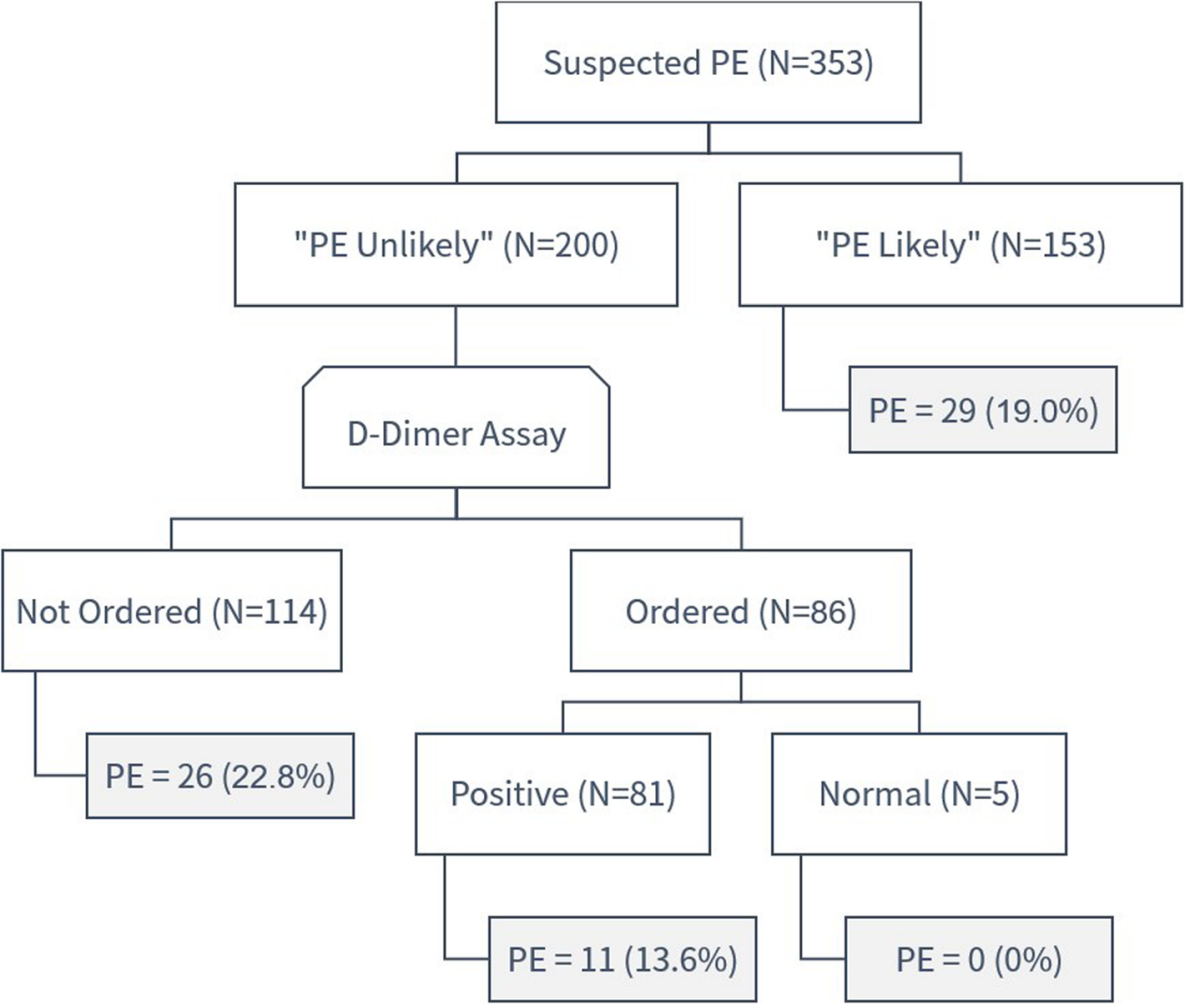
Positive CT-PA rate based on Wells criteria. N number of patients, PE pulmonary embolism

Based on Wells criteria, 200 patients (56.7%) were classified into the “PE unlikely” group and 153 patients (43.3%) in the “PE likely” group (Fig. 1). Interestingly, there were no significant differences between the two groups in terms of age, gender, D-dimer order rate and result, and even the positive CT-PA rate (Table 1).

**Table 1:** Characteristics of the patients in the “PE likely” and “PE unlikely” group

Characteristics		“PE unlikely” (total *n* = 200)		“PE likely” (total *n* = 153)		*χ*^2^ (*P* value)	
		*n*	(%)	*n*	(%)		
Age group (years)	18–30	43	(21.5)	34	(22.2)	3.417	(0.332)
	31–50	83	(41.5)	71	(46.4)		
	51–70	47	(23.5)	24	(15.7)		
	> 70	27	(13.5)	24	(15.7)		
Gender	Male	75	(37.5)	50	(32.7)	0.881	(0.348)
	Female	125	(62.5)	103	(67.3)		
D-dimer assay order	Ordered	86	(43.0)	63	(41.2)	0.118	(0.731)
	Not ordered	114	(57.0)	90	(58.8)		
D-dimer level (mg/dL)	≤ 0.5	5	(5.8)	1	(1.6)	1.681	(0.195)
	> 0.5	81	(94.2)	62	(98.4)		
CT-PA result	Negative	163	(81.5)	124	(81.0)	0.012	(0.914)
	Positive	37	(18.5)	29	(19.0)		

*n* number of patients, CT-PA computed tomography pulmonary angiography

##### “PE likely” group

Out of the 153 patients in the “PE likely” group, 29 patients (19%) had PE including 15 men and 14 women. In 63 patients (41.2%), the CT-PA was performed following D-dimer assay. The D-dimer assay was elevated in all these patients, except for one patient who had normal level and subsequently had a normal CT-PA result.

##### “PE unlikely” group

Among the “PE unlikely” group, the D-dimer was ordered in 86 patients (43%) only. Thus, there were 114 patients (57%) in the “PE unlikely” group who had CT-PA as the first investigation for PE. Of the 114 patients, 88 patients (77.2%) had a negative CT-PA. The CT-PA was performed despite a negative D-dimer for 5 patients from the “PE unlikely” group and none of them had a CT-PA positive for PE. Out of all the CT-PA, 119 CT-PA (33.7%) were requested without D-dimer assay (*n* = 114) or with normal D-dimer level (*n* = 5) despite being in the “PE unlikely” group.

#### PERC criteria

There were 49 patients (13.9%) having negative PERC criteria (Table 2). Only three of these 49 patients had a CT-PA positive for PE, giving a negative predictive value of 93.9%.

**Table 2:** PERC criteria for the patients with suspected PE

1.Item		No	
		*n*	(%)
1	Age ≥ 50 years	230	(65.2)
2	Heart rate ≥ 100	182	(51.6)
3	Oxygen saturation on room air < 95%	275	(77.9)
4	Unilateral leg swelling	335	(94.9)
5	Hemoptysis	338	(95.8)
6	Recent surgery or trauma	318	(90.1)
7	Prior PE or DVT	300	(85.0)
8	Hormone use	346	(98.0)
Overall		49	(13.9)

*PE* pulmonary embolism, *DVT* deep vein thrombosis

## Discussion

Our study revealed that one-third of all CT-PA performed in our institution were not in line with the clinical decision rules. This is because the CT-PA had been requested without having a positive D-dimer level despite the patient being in the “PE unlikely” group. Because the Wells score includes a subjective item which is “an alternative diagnosis is less likely than PE” that has a large contribution of three points to the score, it was initially proposed that the high proportion of unjustified CT-PA scans may be attributed to the retrospective nature of the study. However, we calculated the Wells score again with the assignment of these three points to all patients. Interestingly, the analysis showed that the D-dimer level was not ordered in 18.7% of patients despite being advised by the decision rules, thus, confirming our initial finding.

The reason behind the underutilization of the D-dimer assay is unclear, but the increased patient load and time constraints in the ED might play a role. Furthermore, the use of CT-PA is often justified due to the finding of alternative diagnoses explaining the patient’s clinical presentation, though some evidence did not support this argument [14, 15]. An important contributing factor is the widespread availability of CT with a rapid turnaround time that is available free of charge for all citizens.

Additionally, we had five cases (1.4%) in the “PE unlikely” group who underwent CT-PA despite a normal D-dimer level. Although this seems a very low rate, these were unjustified given the high sensitivity of D-dimer assay which reaches 99% [16]. Notably, none of these cases had PE, thereby confirming the safety of not performing CT-PA scans in these patients.

The finding of inadequate adherence to clinical decision rules is not unique. Several previous studies from different institutions showed comparable results [17–19]. For instance, the National Quality Forum showed that the imaging was avoidable in 32% of patients with suspected PE [20]. A recent study by Kline et al. involving 23 hospitals revealed that out of the 25,870 patients who underwent CT-PA, 59% have not had a D-dimer before the imaging. The same study found a significant positive correlation between the increase in D-dimer assay ordering and the positive CT-PA rate [21].

We identified that 6.1% of patients had PE despite having negative PERC criteria. The PERC criteria were designed to identify patients in whom the risk of unnecessary workup outweighs the risk of PE. A prospective study involving 8138 patients with negative PERC criteria showed that less than 1% had PE [10]. Our finding revealed a relatively high proportion of PE among patients with negative PERC criteria. However, it should be noted that we only had 49 patients with negative PERC criteria. Additionally, Hugli et al. have shown that the prevalence of PE was 5.4% among patients with negative PERC criteria suggesting that the criteria could not safely identify the patients in whom the PE can be excluded without any further workup [22].

The CT-PA remains the first-choice diagnostic modality for the diagnosis of PE because of its high accuracy, especially when incorporated into the clinical decision rules [23]. However, the bedside echocardiography can be more feasible when the rapid or presumptive diagnosis is needed in hemodynamically unstable patients to justify the use of thrombolytic therapy [24]. While the echocardiography may demonstrate the presence of clot or right ventricle strain, it is generally considered to have limited diagnostic value in most cases and it is most beneficial for its prognostic value in patients with confirmed PE [24, 25].

Different strategies have been proposed to increase adherence to the clinical decision rules in the diagnosis of PE. These include the clinical decision support interventions which are based on a computerized physician order entry that prompts the physician to use the Wells criteria when ordering a CT-PA scan. Clinical performance and feedback reports to physicians about their practice and outcome are another intervention to improve the utilization of CT-PA. Formal educational interventions with training sessions have been advised. It has been suggested that the combination of these interventions may have a more significant impact [26].

This study is the first to investigate the utilization of CT-PA in Saudi Arabia. However, the present study has some limitations. The retrospective observational nature of the study is an important limitation. The radiological images were not reviewed for the confirmation of the reported findings. Also, the study did not involve the patients with suspected PE who had not undergone CT-PA. Lastly, the complete reliance on the radiology report for the diagnosis of PE is another limitation given that some studies had shown some concern about the overdiagnosis of clinically insignificant PE [27].

## Conclusion

The study revealed that one-third of all CT-PA was not adhering to the clinical decision rules with significant underutilization of D-dimer assay when indicated. Further studies are needed to explore the barriers leading to inadequate adherence to these rules. To reduce overutilization of imaging, planned interventions to promote the adherence to the current guidelines seem imperative.

## Data Availability

The datasets used and/or analyzed during the current study are available from the corresponding author on reasonable request.

## Abbreviations

CI: Confidence interval
CT-PA: Computed tomography pulmonary angiography
ED: Emergency department
OR: Odd ratio
PERC criteria: Pulmonary embolism rule-out criteria
